# Analysis of Multiple Sarcoma Expression Datasets: Implications for Classification, Oncogenic Pathway Activation and Chemotherapy Resistance

**DOI:** 10.1371/journal.pone.0009747

**Published:** 2010-04-01

**Authors:** Panagiotis A. Konstantinopoulos, Elena Fountzilas, Jeffrey D. Goldsmith, Manoj Bhasin, Kamana Pillay, Nancy Francoeur, Towia A. Libermann, Mark C. Gebhardt, Dimitrios Spentzos

**Affiliations:** 1 Division of Hematology/Oncology, Department of Medicine, Beth Israel Deaconess Medical Center and Harvard Medical School, Boston, Massachusetts, United States of America; 2 Department of Pathology, Beth Israel Deaconess Medical Center and Harvard Medical School, Boston, Massachusetts, United States of America; 3 Genomics Center and Division of Interdisciplinary Medicine and Biotechnology, Department of Medicine, Beth Israel Deaconess Medical Center and Harvard Medical School, Boston, Massachusetts, United States of America; 4 Department of Orthopedic Surgery, Beth Israel Deaconess Medical Center and Harvard Medical School, Boston, Massachusetts, United States of America; Baylor College of Medicine, United States of America

## Abstract

**Background:**

Diagnosis of soft tissue sarcomas (STS) is challenging. Many remain unclassified (not-otherwise-specified, NOS) or grouped in controversial categories such as malignant fibrous histiocytoma (MFH), with unclear therapeutic value. We analyzed several independent microarray datasets, to identify a predictor, use it to classify unclassifiable sarcomas, and assess oncogenic pathway activation and chemotherapy response.

**Methodology/Principal Findings:**

We analyzed 5 independent datasets (325 tumor arrays). We developed and validated a predictor, which was used to reclassify MFH and NOS sarcomas. The molecular “match” between MFH and their predicted subtypes was assessed using genome-wide hierarchical clustering and Subclass-Mapping. Findings were validated in 15 paraffin samples profiled on the DASL platform. Bayesian models of oncogenic pathway activation and chemotherapy response were applied to individual STS samples. A 170-gene predictor was developed and independently validated (80-85% accuracy in all datasets). Most MFH and NOS tumors were reclassified as leiomyosarcomas, liposarcomas and fibrosarcomas. “Molecular match” between MFH and their predicted STS subtypes was confirmed both within and across datasets. This classification revealed previously unrecognized tissue differentiation lines (adipocyte, fibroblastic, smooth-muscle) and was reproduced in paraffin specimens. Different sarcoma subtypes demonstrated distinct oncogenic pathway activation patterns, and reclassified MFH tumors shared oncogenic pathway activation patterns with their predicted subtypes. These patterns were associated with predicted resistance to chemotherapeutic agents commonly used in sarcomas.

**Conclusions/Significance:**

STS profiling can aid in diagnosis through a predictor tracking distinct tissue differentiation in unclassified tumors, and in therapeutic management via oncogenic pathway activation and chemotherapy response assessment.

## Introduction

Soft tissue sarcomas (STS) are a heterogeneous group of mesenchymal tumors traditionally classified according to their morphological resemblance to presumptive cells of origin such as fibroblasts, muscle cells, adipocytes or peripheral nerve-sheath cells [1], [2], [3]. Given their heterogeneity, sarcomas are ideal candidates for molecularly targeted therapies [4], [5]. However, the therapeutic value of current histology-based classification remains unclear. In addition, precise classification is only partially possible, because current histopathologic classification criteria are often inconclusive reflecting the overlapping boundaries between conventional diagnostic groups [6]. This is best exemplified in the case of malignant fibrous histiocytoma (MFH), the second largest subtype by conventional criteria (approximately 20% of cases [7]), a controversial diagnosis which has lately been called in doubt [2], [8]. Furthermore, a significant fraction of STS tumors are unclassifiable, presently called “not otherwise specified” (NOS) [8].

Gene expression profiling has been used in the study of STS [9], [10], [11]. However, these studies were limited by sample size or sample selection, thus clinically applicable diagnostic classification models either have not been reported or have not been independently validated, particularly for the MFH, NOS, and pleomorphic subtypes [12], [13]. Furthermore, the therapeutic utility of their findings was limited by the inability to predict activation status of relevant oncogenic pathways in a given individual tumor specimen, as opposed to presenting average expression patterns in predefined tumor subgroups. In order to address these challenges, we integrated five publicly available microarray datasets originating from different laboratories around the world, to develop and validate a class predictor, which we then used to molecularly characterize and reclassify MFH and uncategorized sarcomas. Further, we validated these findings in a new group of paraffin derived tumors. Finally, in order to assess the therapeutic relevance of genomic classification, we used computational models to determine the activation status of oncogenic pathways, for which specific targeted inhibitors are in clinical development in sarcoma, and studied the association of pathway activation with histologic subtype and chemoresponse.

## Results

### Development of a multi-gene predictor in the training dataset

**Figure 1:**
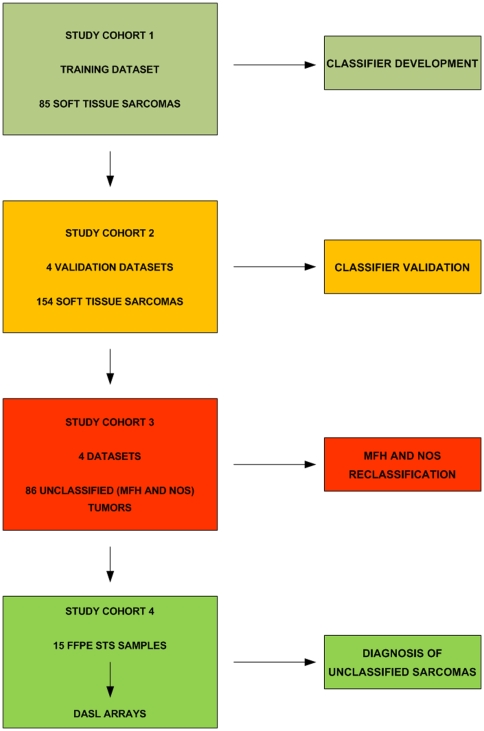
Consort Diagram (Study Design). A multi-class gene expression predictor for 6 major histologic subtypes* was developed in the training dataset (study cohort 1) and validated in 4 independent datasets (study cohort 2). The predictor was used to reclassify MFH (Malignant Fibrous Histiocytoma) and NOS (Not Otherwise Specified) tumors (study cohort 3) into known subtypes. The predictor's performance and capacity to classify unknown type sarcomas were also validated in paraffin STS samples (study cohort 4). * Liposarcoma (LIPO), Leiomyosarcoma (LEIO), Fibrosarcoma (FBR), Malignant Peripheral Nerve Sheath Tumor (MPNST), Synovial Sarcoma (SYN), Rhabdomyosarcoma (RHAB).

**Figure 2:**
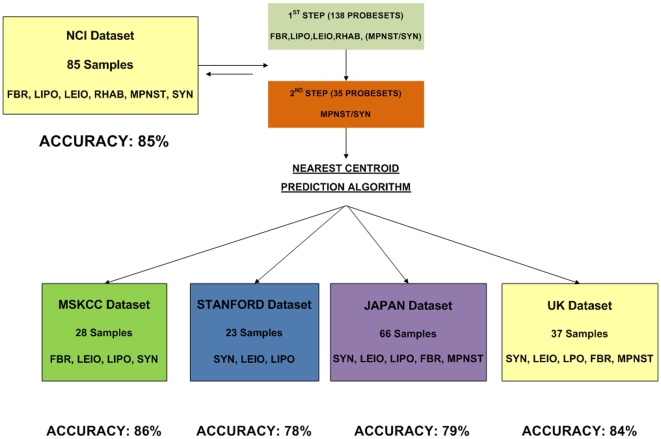
Predictor development and validation. A two-step 6-class predictor was identified in the NCI dataset and validated in the remaining four datasets. First step: A 138-gene model classifies LEIO, LIPO, FBR, RHAB and a composite class including MPNST and SS. Second step: The composite class is separated into MPNST and SYN tumors using a 35-gene model.

We used study cohort 1 (NCI, the largest dataset including all 6 aforementioned subtypes) in order to train a nearest centroid classifier of the 6 subtypes (LEIO, LIPO, RHAB, MPNST, SYN and FIBRO) frequently presenting differential diagnosis problems, (Figure 1). A single step 6-class model could not be developed because of the significant gene expression overlap between MPNST and SYN classes. Therefore a two-step classifier was defined (Figure 2). In the first step, a 138-gene model distinguished LEIO, LIPO, FIBRO, RHAB and a composite class including MPNST and SYN. In the second step, the composite class is separated into MPNST and SYN tumors using a 35-gene model. Due to a partial gene overlap between the two-step predictors the combined model included 170 genes.

**Figure 3:**
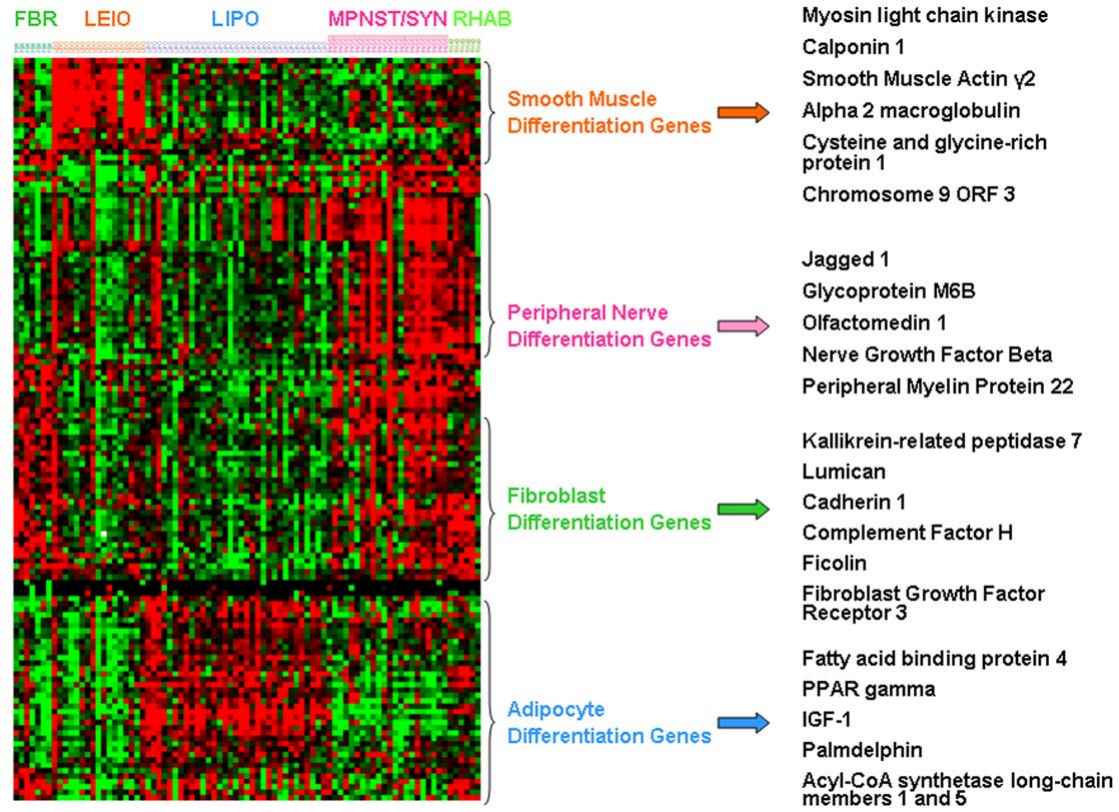
Distinct expression patterns of the first step 138-gene predictor in the NCI dataset. Selected predictor genes associated with distinct differentiation states (smooth muscle, peripheral nerve, fibroblast and adipocyte differentiation) based on Gene Ontology or literature evidence.

**Figure 4:**
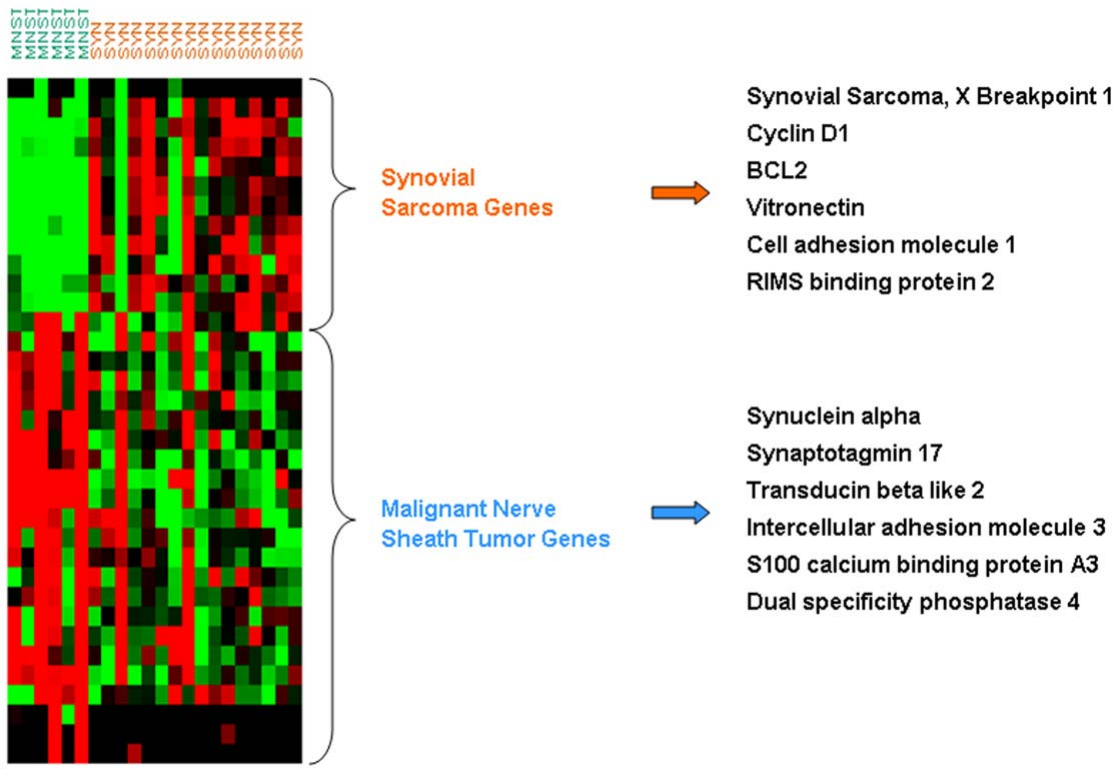
Distinct expression patterns of the second step 35-gene classifier in the NCI dataset. Selected genes overexpressed in synovial and malignant peripheral nerve sheath tumors, are shown on the right, representing potential novel tissue differentiation markers.

This optimal predictor was 85% accurate for the 6 classes (Figure 2 and Table S1). The genelists of the 1^st^ and 2^nd^ step predictors are shown in Tables S3 and S4. The distinct expression patterns of the first step and second step classifiers in the NCI dataset are displayed in Figures 3 and 4 respectively. Detailed training accuracies for all classes for the nearest centroid predictor are shown in Tables S1 and S2.

Importantly, the predictor included several genes associated with distinct differentiation states (i.e. fibroblastic, smooth muscle, adipocytic and peripheral nerve differentiation) (Figure 3). Appropriately, these genes were overexpressed in the corresponding subtypes.

### The 170-gene predictor accurately classifies STS subtypes in four independent datasets

We mapped the 170-gene set across the different platforms of the 4 datasets in study cohort 2, and despite the many technical differences among them we were able to reproduce its performance. Specifically, its accuracy was 86%, 78%, 79%, and 84% in the MSKCC, Stanford, Japan and UK datasets, respectively by leave one out cross validation (permutation p<0.001 in all cases) (Figure 2, Table S1). Detailed accuracy, sensitivity and specificity for each class in the training and validation datasets are shown in Tables S1 and S2. Due to platform differences, a more direct validation of the predictor accuracy was only possible among the 3 U 133 datasets, after allowing for gene content mismatch compared with the NCI cDNA original predictor. Thus, training the (modified) predictor on each of the U 133 datasets and applying it to the other U 133 datasets, we obtained 70–75% accuracy.

### Reclassification of MFH and NOS samples using the 170-gene STS predictor

We used the 170-gene predictor in study cohort 3, to reclassify 76 MFH and 10 NOS samples. As noted, the MFH and NOS samples were not used in the development or validation of the predictor.

The majority (68 out of 76) MFH tumors were predicted as liposarcomas (46%-35 samples), fibrosarcomas (29%-22 samples) and leiomyosarcomas (14%-11 samples), while 7 out of 10 NOS tumors (in the NCI dataset) were also predicted as liposarcomas (3), leiomyosarcomas (3) and fibrosarcomas (1). The remaining MFH and NOS tumors (11 samples) were predicted as malignant peripheral nerve sheath tumors (4 samples), synovial sarcomas (6 samples) and rhabdomyosarcoma (1 sample).

### ‘Molecular match’ between reclassified MFH and NOS samples and their corresponding STS subtypes

We reasoned that if our proposed MFH reclassification using the 170-gene predictor is valid, it should reflect the overall molecular similarity between reclassified MFH and their corresponding subtypes, above and beyond the prediction by the 170-gene predictor. We addressed this question both within each as well as across datasets.

### a. Molecular match by clustering within each dataset

**Figure 5:**
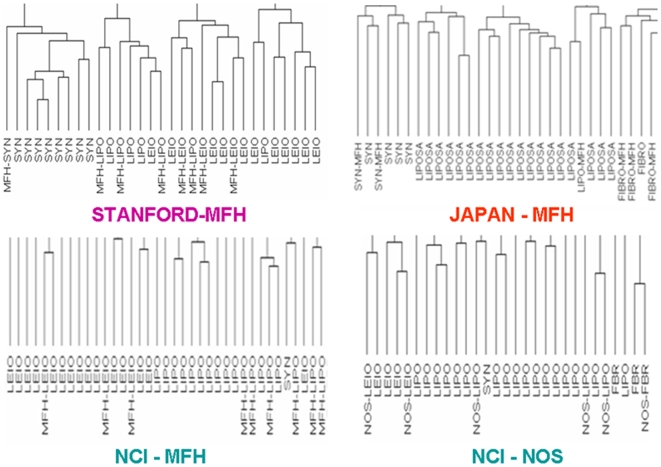
Assessment of MFH/NOS reclassification within each dataset. Genome-wide hierarchical clustering reveals that MFH samples predicted as LIPO, LEIO, and SYN, clustered together with conventional LIPO, LEIO and SYN samples respectively. Similarly, NOS samples reclassified as liposarcomas leiomyosarcomas and fibrosarcomas clustered together with conventional LIPO, LEIO and FBR samples respectively. For Stanford dataset, the complete dendrogram is presented here. For the remaining datasets, due to size limitations, representative portions of the dendrograms are shown. Full dendrograms are shown in Figure S2, which also demonstrate that 24% of MFH samples (in total) did not cluster with the predicted subtypes.

We performed unsupervised hierarchical clustering and assessed whether the MFH samples preferentially grouped with samples from their predicted subtype using the top-33% variant genes, i.e. 4200, 7428, 922 probe-sets in the NCI, Japan and MSKCC, and Stanford datasets, respectively. Indeed, 58 out of the 76 reclassified MFH samples (76%) clustered together with samples from their predicted STS subtypes suggesting that the 170-gene predictor reflects an overall ‘molecular match’ between them. Figure 5 and Figure S2 show hierarchical clustering of the four datasets, where MFH samples reclassified as LIPO, LEIO, FIBRO and SYN, clustered with conventional LIPO, LEIO, FIBRO and SYN samples respectively. The specific clustering results for the classification of MFH samples are presented in Table S7.

Furthermore, MFH-samples predicted as LIPO (MFH-LIPO) did not cluster exclusively with myxoid or with non-myxoid liposarcomas; rather certain MFH-LIPO clustered with myxoid while other MFH-LIPO clustered with non-myxoid liposarcomas suggesting that our predictor is capturing information associated with adipocyte differentiation irrespective of the myxoid or non-myxoid subclassification (Figure S1).

The same analysis was performed for the NOS sample predictions in the NCI dataset, and 6 out of the 10 NOS samples clustered with their predicted subtypes (Figure 5).

### b. Molecular match by Subclass Mapping across different datasets

**Figure 6:**
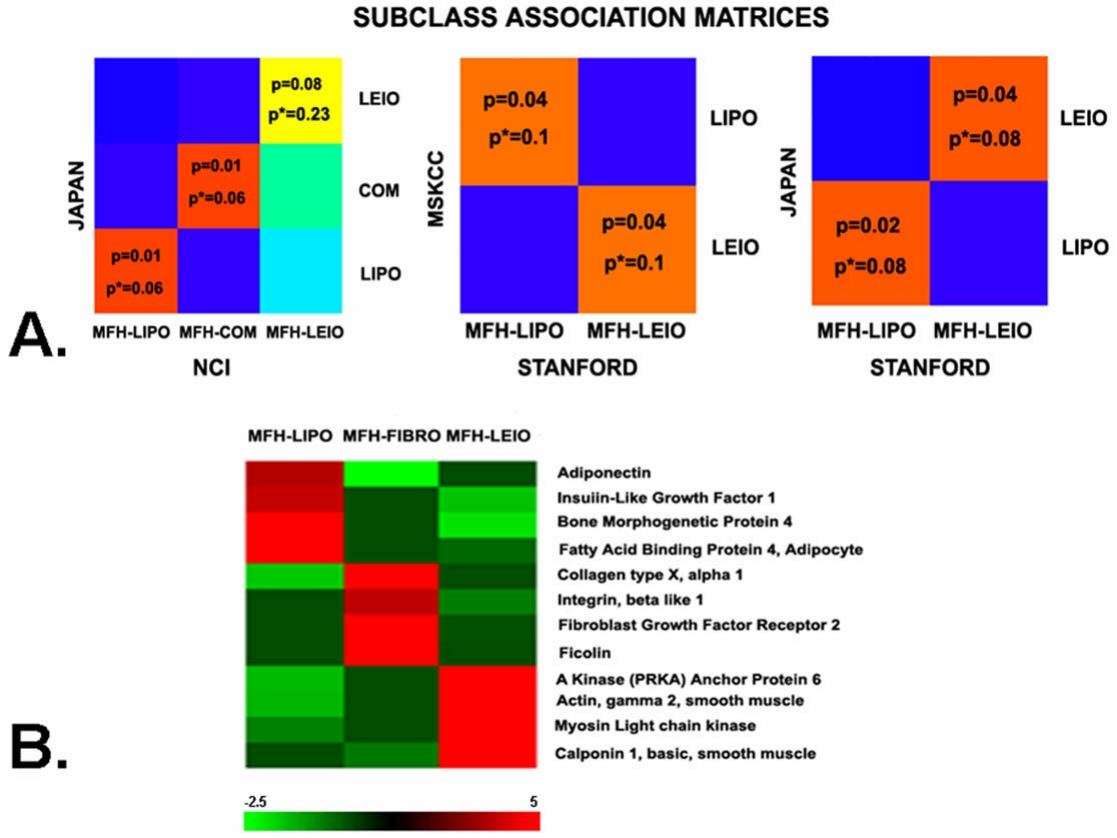
Reclassified MFH samples correspond to their respective STS subtypes across different datasets and express the tissue specific markers of their respective STS subtypes. **A) Subclass Association Matrices assessing molecular correspondence of reclassified MFH samples across different datasets:** ***Left***: NCI versus Japan dataset (COM: composite MPNST-SYN group). ***Center*** *:* Stanford versus MSKCC dataset. ***Right**:* Stanford versus Japan dataset. In all cases p* is Bonferroni-corrected. (**Color scale**: Red and Yellow colors indicate p<0.05 and 0.05≤p<0.1, respectively suggesting strong molecular correspondence between subtypes in different datasets. Other colors indicate p>0.1 suggesting lack of molecular correspondence). Details are provided in Table S5). **B) Expression of tissue specific markers in MFH samples**: Heat map showing upregulation of selected genes associated with smooth muscle, fibroblast and adipocyte differentiation (based on Gene Ontology or literature) in MFH tumors predicted as leio-, lipo-, or fibrosarcoma, respectively, compared to the rest of MFH tumors (t-test p<0.05). **Color scale** (saturated at 5-fold upregulation) indicates average fold change for each predicted MFH subclass compared to the rest of MFH tumors. Detailed fold changes are provided in Figure S3.

To strengthen the molecular relevance of the MFH reclassification we investigated whether MFH samples were molecularly similar with samples from their predicted STS subtype across different datasets. To achieve this, we used the Subclass Mapping (SubMap) methodology, specifically developed to assess the commonality of subtypes/subclasses in independent and disparate datasets (a candidate subclass is included in the analysis only if it contains at least 10% of all the samples of a dataset [14]). As shown in Figure 6 (upper panel), MFH samples from the NCI and Stanford datasets matched their predicted subtypes from the MSKCC and Japan datasets despite their many technical differences. Because of small sample size, this analysis could not be performed for NOS tumors.

### Reclassified MFH tumors appropriately overexpress genes associated with distinct differentiation lines

To further demonstrate the molecular basis of reclassifying MFH, we examined whether MFH samples overexpressed genes associated with their predicted differentiation lines. Indeed, MFH tumors predicted as liposarcomas (MFH-LIPO) overexpressed genes associated with adipocyte differentiation compared to the rest of the MFH tumors. Similarly, MFH tumors predicted as leiomyosarcomas (MFH-LEIO) overexpressed genes associated with smooth muscle differentiation and MFH sarcomas predicted as fibrosarcomas (MFH-FIBRO) overexpressed genes associated with fibroblast differentiation (Figure 6). We could not reliably assess specific marker expression for MFH-MPNST, and MFH-SYN given the small number of tumors predicted as these categories. Figure S3 displays the fold upregulation of selected genes associated with smooth muscle, adipocyte and fibroblast differentiation in MFH tumors predicted as leio-, lipo- and fibrosarcomas.

### Utility of the STS predictor in unclassifiable paraffin sarcoma specimens

We next evaluated the ability of our STS predictor to reclassify formalin fixed paraffin embedded NOS samples in order to assess its broader applicability for clinical practice and future large scale clinical research. These NOS samples had been previously evaluated by a sarcoma pathology expert (J.G) using state of the art current histopathologic methodology and could not possibly be classified into any of the known STS types.

Before applying our predictor to the unclassified samples, we verified its accuracy in 10 STS samples with known diagnosis. We trained the predictor (modified due to partial gene content mismatch) on the combined U 133 datasets and directly applied it on the independent DASL paraffin gene expression dataset. The STS classifier accurately predicted 8 of the 10 samples, demonstrating accuracy identical to that previously estimated in the 5 public frozen-tissue based datasets, thus validating its performance in samples with known diagnosis and in paraffin tissue. We then directly applied our predictor to the 5 unclassifiable (NOS samples) within the paraffin cohort, and 4 of them were classified as liposarcomas and 1 as leiomyosarcoma. We then examined expression of tissue specific genes in the 4 NOS samples classified as LIPO, and found that they appropriately overexpressed genes associated with adipocyte differentiation including adiponectin, insulin-like growth factor 1, and adipocyte fatty acid binding protein 4 (3.1 fold, 2.4 fold, and 1.5 fold upregulated (t test p = 0.06, 0.15 and 0.07 respectively), respectively, as compared to the known non-LIPO samples, (3 LEIO, 2 SYN and 2 MPNST). These findings confirm the utility of the classifier in real time and routinely collected paraffin sarcoma samples and its capacity to detect previously unrecognized tissue differentiation lineage in truly unclassified sarcoma tumors.

### Unique patterns of oncogenic pathway activation in STS subtypes

In order to evaluate whether STS classification bears potential biologic or therapeutic implications, we estimated the probability of activation of known oncogenic pathways in individual samples, using validated gene expression “read outs” previously generated in vitro as a result of controlled experimental activation of these pathways. We focused on Src, Ras and PI3K pathways, for which pharmacologic inhibitors are currently in clinical development in sarcoma, and we assessed tumors from the 3 Affymetrix oligonucleotide U133A datasets (Japan, MSKCC and UK datasets) in our study. Since these gene expression models of pathway activation were generated using oligonucleotide Affymetrix arrays, and most Affymetrix probesets included in these predictors were not present in the cDNA datasets, we did not assess pathway activation in tumors from the 2 cDNA datasets as these predictions would have been less reliable.

The probability of activation of the Src, Ras and PI3K pathways was statistically significantly different between different subtypes (Kruskal-Wallis p<0.001, p = 0.002, p = 0.021 respectively). More specifically, FIBRO demonstrated higher probability of Ras and PI3K pathway activation (Mann-Whitney p = 0.044 and p = 0.013 respectively) and LIPO demonstrated higher probability of Src pathway activation (p<0.001) and lower probability of PI3K pathway activation (p = 0.06) compared to the rest of the samples (Table S8). Conversely, synovial sarcomas were associated with statistically significantly lower probability of Src and Ras pathway activation compared to the rest of the samples (p = 0.005 and p<0.001 respectively). Finally, LEIO samples did not show any particular pattern of activation of any of the Src, Ras or PI3K pathways (Table S8).

### Reclassified MFH share similar patterns of oncogenic pathway activation with their corresponding subtypes

In order to assess whether MFH reclassification using our 170-gene predictor is tracking specific oncogenic pathway activation patterns, we evaluated the activation status of Src, Ras and PI3K pathways in the 30 MFH samples of the U133 datasets using the aforementioned gene expression “readouts”. Similar to their predicted STS subtypes, MFH sarcomas predicted as fibrosarcomas (MFH-FIBRO) had similarly high average probability of PI3K pathway activation (0.99 vs 0.99 in MFH-FIBRO and FIBRO respectively) and similarly low average probability of Src pathway activation (0.01 vs 0.13 respectively). Furthermore, MFH sarcomas predicted as liposarcomas (MFH-LIPO) had similar average probability of PI3K pathway activation (0.75 vs 0.73 in MFH-LIPO and LIPO respectively). Similar to LEIO, MFH-LEIO did not demonstrate any specific pattern of pathway activation compared to the rest of the samples. There were only two MFH samples predicted as synovial, so no firm conclusions could be reached for this subset.

### Distinct patterns of oncogenic pathway activation are associated with chemotherapy resistance

Given the modest predictive value of histology for chemotherapy response, we investigated whether patterns of oncogenic pathway activation are associated with resistance to commonly used chemotherapy agents in sarcoma. For this purpose, we used gene expression readouts (previously developed using Affymetrix arrays from the NCI 60 cancer cell line panel) predicting the probability of resistance to adriamycin, docetaxel and cyclophosphamide, (which was used as a surrogate for ifosfamide, for which NCI 60 resistance data were not available). The probability of resistance to each chemotherapy drug was estimated for each individual sample included in the U133 datasets of our study. As with pathway activation predictions, we did not assess the possibility of chemotherapy resistance in tumors from the two cDNA datasets as these predictions would have been less reliable (most Affymetrix probesets from these predictors were not present in the cDNA datasets).

**Figure 7:**
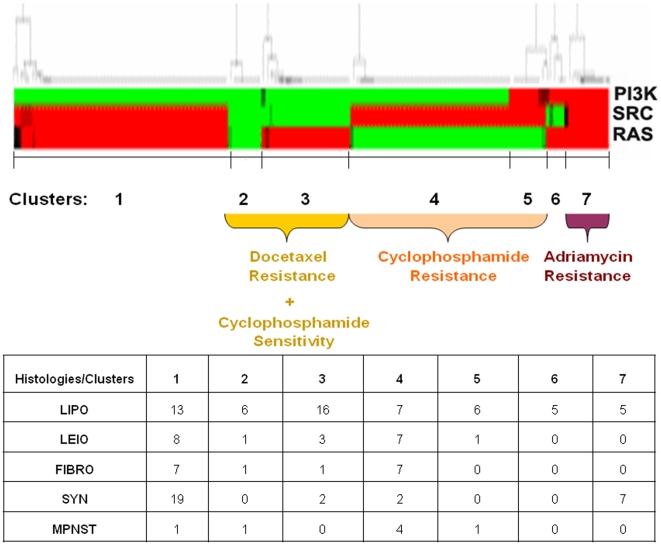
Association between patterns of oncogenic pathway activation and resistance to chemotherapy drugs. Unsupervised hierarchical clustering of 161 tumor samples based on individual sample probability of Src, Ras and PI3K pathway activation reveals 7 clusters with distinct patterns of pathway activation and association with chemotherapy resistance: Cluster 7 is associated with higher probability of adriamycin resistance (p = 0.002), clusters 4 and 5 are associated with higher probability of cyclophosphamide resistance (p = 0.01), and clusters 2 and 3 are associated with higher probability of docetaxel resistance (p = 0.01), compared to the rest of the samples. The composition of STS histologies in each cluster is also presented.

We then performed hierarchical clustering of the samples based on each sample's probability of activation of Src, Ras and PI3K pathways, and observed that samples were classified into seven clusters with distinct pathway activation patterns. As shown in Figure 7, different patterns of pathway activation were associated with resistance to different chemotherapy agents. Cluster 7 (Src, Ras and PI3K activation) was associated with higher probability of adriamycin resistance (Mann-Whitney p = 0.002), clusters 4 and 5 (Src with or without PI3K activation) were associated with higher probability of cyclophosphamide resistance (p = 0.01) and clusters 2 and 3 (Ras or no pathway activation) were associated with higher probability of docetaxel resistance (p = 0.01) compared to the rest of the samples. Finally, we examined the distribution of the different histologies within the pathway clusters and found that it was random with the exception of liposarcomas being overrepresented (16 out of 58) in cluster 3 and synovial sarcoma being overrepresented in cluster 1 (19 out of 30). However, these clusters did not recapitulate any previously reported associations with chemotherapy response patterns.

### Gene expression patterns of tumors with activated Ras pathway are enriched for targets of the let-7 miRNA family

It has been postulated that expression profiles may partly be surrogates for microRNA alterations in sarcoma. Previous in vitro data indicate that Ras is regulated by the let-7 microRNA family [15]. In this regard, we tested the hypothesis that the gene expression pattern of clinical samples with predicted activation of Ras pathway, were enriched for gene targets of the let-7 microRNA family. We performed microRNA target gene set enrichment analysis in samples with or without predicted activation of Ras pathway using the functional scoring method. Indeed, we found that the gene expression pattern of the samples with predicted Ras pathway activation was enriched for targets of all microRNAs of the let-7 family (p<0.05 for all miRNAs of the let-7 family in all 3 datasets – Table S6), suggesting that let-7 may play a role in Ras activation in human sarcoma tumors.

## Discussion

Soft tissue sarcomas are heterogeneous neoplasias, thus conceptually well-suited for application of targeted therapies [4], [5], [16]. Development of such therapies has been limited by the fact that traditional histopathologic classification has never been shown to carry substantial therapeutic value. This may be partly related to the additional challenge that current histomorphologic classification criteria are frequently inconclusive and do not fully capture the underlying molecular complexity of these tumors, leaving a sizable fraction of them unclassified or grouped in controversial entities, such as MFH [2], [6], [8], [17]. Previous microarray studies have analyzed gene expression patterns [12], [18], [19] yielding evidence of substantial differences among sarcoma subtypes. However, most of the findings were not replicated, and the single previously reported diagnostic predictor was not independently validated [13], thus being subject to limitations related to overfitting [20], [21] and single-study bias. Furthermore, despite its theoretical promise, the therapeutic relevance of proposed genomic classification of sarcomas has been difficult to assess, (particularly for those types not associated with a targetable “necessary and sufficient” molecular abnormality), partly because of the inability to predict activation status of multiple relevant oncogenic pathways in a given individual tumor specimen.

In this study, we integrated 5 publicly available sarcoma microarray datasets [12], [13], [18], [19], [22], with the intent to address current diagnostic as well as therapeutic challenges in sarcoma management. We, first, identified a 170-gene classifier of six major subtypes (Figure 2) that frequently pose differential diagnosis problems, as they can all present with pleomorphic poorly differentiated variants. This predictor was validated in four independent datasets derived from different laboratories in different parts of the world. Despite the significant inter-laboratory and platform differences (oligonucleotide versus cDNA arrays), the classifier demonstrated 78-86% accuracy across all 4 independent datasets (Figure 2). The classifier included several genes associated with distinct differentiation states (i.e. fibroblastic, myogenic, adipocytic and neural differentiation) (Figure 3) suggesting that assignment of a sample to a specific class reflects its molecular resemblance to differentiated mesenchymal cells such as fibroblasts, muscle cells, adipocytes or peripheral nerve-sheath cells, a resemblance that is otherwise not appreciated by standard histopathologic criteria. Several classifier genes are potential drug development targets, for instance the IGF-1 [23], PPAR gamma [24], NGF beta [25] and FGF receptor 3 genes [26]. While the biologic program responsible for the behavior of sarcomas is likely to encompass a much larger number of gene networks, these observations suggest that the relatively limited classifier gene set may still capture therapeutically relevant mechanisms.

A particular challenge for sarcoma classification remains the elusive nature of “MFH” sarcomas. Microarray studies indicated that MFH tumors comprise a complex group not forming a distinct cluster, raising the possibility that MFH does not represent a unique molecular category [12]. Also, previous morphologic studies have suggested that MFH tumors share similarity with pleomorphic variants of other known subtypes [2], [17]. While it has been suggested by the WHO classification, that the terminology “MFH” will be abandoned when criteria for reclassification of pleomorphic sarcoma can be reproducibly defined [2], [8], currently no such criteria exist, and the similarity between MFH and other subtypes in these studies was determined by a semi-quantitative histopathologic stains without regard to genome-wide molecular resemblance [2], [17]. Of note, MFH tumors with myogenic differentiation behave more aggressively indicating that MFH reclassification may also be prognostically useful [27].

We showed that our validated multi-gene predictor can reclassify MFH and other uncharacterized tumors into one of the major subtypes, the majority predicted as leiomyosarcomas, liposarcomas and fibrosarcomas. The validity of this reclassification was confirmed by sophisticated bioinformatics approaches (Figures 5, 6) including a recently developed method (SubMap), uniquely suitable to assess the resemblance of subtypes identified in multiple, independent, and technically disparate datasets [14]. Furthermore, by examining the expression profiles of the MFH samples, we identified a number of tissue differentiation genes above and beyond the 170-gene predictor that were also appropriately overexpressed according to their predicted histology. In the absence of another “gold standard” metric, the finding of tissue specific genes over expressed in previously unclassified tumors in accordance to their predicted subtype, serves as confirmation of the classification potential of our predictor. Of note, for liposarcomas, we were able to show that our predictor is tracking adipocyte differentiation of MFH irrespective of the myxoid or non-myxoid sub-classification. Finally, but no less importantly, we again validated the performance on the predictor in real life paraffin specimens, including its capacity to reveal tissue lineage in specimens that are currently impossible to classify with state-of-the-art histopathologic examination. These findings, taken together, suggest that our proposed reclassification is not merely a mathematical model function; rather it is tracking real underlying molecular sarcoma “phenotypes” based on tissue differentiation lines. Our analysis supports the concept, proposed originally by Fletcher [17], that MFH represents the end stage of dedifferentiation of many sarcoma subtypes rather than a distinct entity.

It should be noted, however, that it is possible that certain MFH/NOS tumors may be so undifferentiated that they may not harbor any distinct lineage. In this case, our predictor would result in “overclassification” of these samples, forcing them into one of the conventional classes. The extent of this error is not possible to know with certainty. The fraction of MFH samples that did not co-cluster with their predicted subtype in Figure 5 (24%) may be an estimate suggesting that the subset of “overclassified” MFH tumors is small.

The second aim of our study was to explore whether genomic reclassification of sarcomas using our predictor bears biologic and therapeutic implications. Several oncogenic pathway inhibitors are currently undergoing clinical trial evaluation in sarcoma including drugs targeting the PI3K/mTOR (deferolimus and everolimus), Src (dasatinib and AZD0530), and the Ras/Raf pathway (sorafenib). However, the activation status of these pathways in individual sarcoma samples and across different histologic subtypes has not been possible to determine, making it difficult to prioritize patients for targeted therapies. For this purpose, we applied previously validated gene expression “read outs” of oncogenic pathway activation to individual samples, and discovered that different subtypes demonstrate distinct patterns of activation of these oncogenic pathways. Interestingly, reclassified MFH demonstrated similar patterns of oncogenic pathway activation as their corresponding predicted subtypes, providing further evidence that the 170-gene predictor reflects the overall molecular program in sarcomas, with therapeutic implications.

Although we showed an association between different histologies and patterns of oncogenic pathway activation, it is also well-known that the association between histology and chemotherapy resistance is only modest, and in many cases unproven, in soft tissue sarcomas. Our findings suggest that oncogenic pathway activation patterns, transcending histologic classes, and assessed by gene expression “read outs”, may serve as useful predictors of resistance to chemotherapy drugs commonly used in sarcoma (Figure 7), perhaps overriding previously considered modest associations between histology and chemoresistance. Although proof of an etiologic association between specific patterns of oncogenic pathway activation and chemotherapy resistance, or *in vitro* demonstration of novel chemoresistance mechanisms were beyond the scope of this study, our findings reveal interesting therapeutic research strategies that can be studied in properly designed prospective studies. For example, prior knowledge of oncogenic pathway activation patterns in individual sarcoma samples may aid in prioritizing patients for novel molecularly targeted agents, conventional chemotherapeutic agents, or combinations thereof. However, our study was limited by the lack of clinical data (i.e. chemotherapy response or outcome data) linked with the public microarray datasets we used, a fact that prevented us from being able to further test the chemoresistance patterns identified in our analysis.

Finally, the finding that the gene expression patterns of Ras-activated tumor samples are enriched for gene targets of the let-7 microRNA family, consistent with compelling experimental evidence of Ras regulation by let- 7 miRNAs [15], raises the possibility that microRNA alterations may contribute to oncogenic pathway activation, and is consistent with the concept that gene expression patterns may be partly surrogates for microRNA alterations [28], [29], [30] in primary sarcoma tumors.

Our study demonstrates the power of integrated analysis of multiple and diverse microarray datasets, in order to generate and validate clinically useful models and concepts, in a cost effective manner. We identified a multi-gene STS predictor, reproduced for the first time in multiple independent gene expression datasets, and in routinely collected paraffin tissue, which could serve as an aid to standard histopathologic methods, especially in the diagnosis of pleomorphic tumors that are impossible to classify based on state of the art histopathology. Our findings support the concept that MFH and unclassified (NOS) sarcomas can be reclassified into existing sarcoma subtypes, and proposes a tool for clinical application that reflects previously unrecognized lines of sarcoma differentiation. Finally, our results support the notion that genomic classification may carry potential therapeutic implications, and provide novel therapeutic research hypotheses regarding individualization of targeted therapies and overcoming chemotherapy resistance in soft tissue sarcomas.

## Materials and Methods

### Assembly and processing of Gene Expression Datasets

Our study included five public microarray datasets [12], [13], [18], [19], [22] (Table 1, Figures 1 and 2) with 325 tumors of the six STS subtypes [liposarcomas (LIPO), leiomyosarcomas (LEIO), rhabdomyosarcomas (RHAB), malignant peripheral nerve sheath tumors (MPNST), synovial sarcomas SYN and fibrosarcomas (FIBRO)] that frequently present differential diagnosis problems. Red/green channel expression data were retrieved from 2 cDNA datasets (NCI, Stanford) and raw data were retrieved from 3 oligonucleotide Affymetrix U133A datasets, (UK, Japan, MSKCC). cDNA data were normalized using the median normalization method and Affymetrix .CEL file data were processed using the Robust Multi-Array Average (RMA) algorithm [31]. Analyses described below were performed using the BRB Array Tools package (Dr Richard Simon, NCI), unless noted otherwise.

**Table 1:** Content of the 5 microarray expression datasets.

DATASETS	PLATFORM	SAMPLES	HISTOLOGIES
*NCI*	cDNA	133	SYN (16), LEIO (17), LIPO (33), MPNST (6), FIBRO (7), RHAB (6), **MFH (38)**, **NOS (10)**
*STANFORD*	cDNA	31	SYN (8), LEIO (11), LIPO (4), **MFH (8)**
*UK*	U133A	37	SYN (10), LEIO (8), LIPO (10),MPNST (4), FIBRO (5)
*JAPAN*	U133A	87	SYN (16), LEIO (6), LIPO (37),MPNST (3), FIBRO (4), **MFH (21)**
*MSKCC*	U133A	37	SYN (4), LEIO (6), LIPO (11),FIBRO (7), **MFH (9)**
*TOTAL PATIENTS*		325	SYN (54), LEIO (48), LIPO (95), MPNST (13), FIBRO (23), RHAB (6), **MFH (76)**, **NOS (10)**

### RNA Isolation from paraffin specimens and Illumina Whole Genome DASL array hybridization

Paraffin specimens from 15 soft tissue sarcomas including 5 NOS were cut into 1-3 mm cores at the BIDMC Histology Core facility. These included 10 paraffin STS samples with known diagnosis (3 LIPO, 3 LEIO, 2 SYN and 2 MPNST) and 5 paraffin NOS samples that had been previously evaluated by a sarcoma pathology expert (J.G) using state of the art current histopathologic methodology and could not possibly be classified into any of the known STS types. These samples, all archived between 2003 and 2006 at the Beth Israel Deaconess Medical Center Pathology Department, were chosen on the basis of tissue availability and adequate RNA yield for microarray studies. IRB approval for tissue utilization was obtained as per standard institutional protocols. Total RNA was isolated using the Qiagen RNeasy formalin-fixed, paraffin-embedded (FFPE) protocol according to the manufacturer's instructions. Whole genome DASL (cDNA-mediated, Annealing, Selection, and Ligation) arrays (Illumina, CA), containing 24,000 gene transcripts were used to profile the paraffin specimens on an Illumina BeadStation. The DASL array experiments were carried out at the Children's Hospital (Boston) Microarray Core facility as per manufacturer's instructions and as previously described [32], [33].

### Classification analysis design

Figure 1 shows our study workflow. We defined 4 “study cohorts”. Study cohort 1 (NCI) was used to optimize a gene expression predictor. Study cohort 2 (4 datasets – Stanford, UK, Japan, MSKCC – not used in step 1) was used to independently validate the predictor. Study cohort 3 (MFH and NOS samples from all datasets, not used in prior steps) was used to reclassify previously uncategorized sarcomas based on the predictor. Finally, this predictor was applied to study cohort 4, which consisted of 15 paraffin STS samples that were profiled using whole-genome DASL (cDNA-mediated, Annealing, Selection, and Ligation) arrays. In this step, we used the predictor to classify NOS samples that were impossible to classify using current, state of the art histopathologic evaluation and staining.

### Development and validation of a multi-gene predictor

Using the Nearest Centroid algorithm [34], [35] we developed a predictor of 6 subtypes on the NCI dataset –the largest and only one that included all six subtypes. We trained the classifier selecting genes differentially expressed between classes by F-test. Classifier accuracy and statistical significance were assessed using leave-one-out cross-validation and a random permutation test to control for over-fitting. The best-performing training classifier included 138 genes (F test cut off, p<4×10^−7^) and demonstrated an accuracy of 85%. Sensitivity analysis using an F-test threshold from p<10^−7^ (100 genes) to p<5×10^−7^ (160 genes) demonstrated only minimal loss of performance with accuracy of 79–82%. This classifier was mapped across different platforms using Affymetrix annotation files and applied to the four independent public datasets as well as the paraffin based dataset. The overlap of predictor genes across different platforms was as follows: i) Among the NCI cDNA platform and the 3 U133 datasets there was complete overlap of the 1^st^ and 2^nd^ step predictor genes (i.e. 138 genes and 35 genes respectively), ii) Between the NCI cDNA platform and the DASL Illumina (paraffin) dataset the overlap was 136 genes for the 1^st^ and 34 for the 2^nd^ step predictor) and iii) Between the NCI cDNA platform and the cDNA (Stanford) dataset the overlap was 62 genes for the 1^st^ and 19 genes for the 2^nd^ step predictor).

Allowing for partial loss of classifier genes and the technical disparity between the different platforms, classifier accuracy was further assessed using leave one out cross validation and a random permutation test in each of the 4 independent public datasets. Further, for a more direct validation of accuracy, we also collated the 3 U133 datasets into one combined dataset after adjusting for platform effect using empirical Bayes method [36]. Then, we trained a modified predictor (using the same genes but creating a different model due to technical platform differences) in the largest U133 dataset (JAPAN), and directly applied it to the remaining datasets (UK and MSKCC) and vice versa. Finally, for prediction of the paraffin samples we collated all 4 datasets (3 U133 and the DASL paraffin dataset) after adjusting for platform effect using empirical Bayes method [36]. The final predictor (modified due to partial gene content mismatch with the U133 platform) was trained on the 3 combined U 133 datasets and directly applied to the DASL dataset.

### MFH and NOS reclassification using the multi-gene predictor

We used the predictor to reclassify the 76 MFH and the 10 NOS samples into the 6 subtypes after mapping the predictor genes across different platforms (from cDNA to Affymetrix U133) as above. No mapping was necessary for the NOS samples that were all contained in the NCI dataset.

### Validation of MFH and NOS reclassification within each dataset using genome-wide hierarchical clustering

In order to assess whether our MFH reclassification reflected true molecular similarity of the reclassified MFH samples with their corresponding STS subtypes, we performed unsupervised hierarchical clustering of all tumors (including MFH samples) within each dataset using the complete linkage method and the one minus centered correlation as a distance metric [37]. A large number of genes (top 33% variant) were included in this analysis in order to overcome the overfitting bias of the optimized 170-gene predictor. Then, we assessed whether each reclassified MFH sample clustered together with the samples of its corresponding STS class (the class it had been reclassified into) and repeated the same process for the NOS reclassification.

### Validation of MFH reclassification across different datasets using Subclass Mapping (SubMap)

Hierarchical clustering cannot assess molecular correspondence between phenotypes across different datasets. Thus, we examined the molecular similarity between reclassified MFH samples and their predicted corresponding STS subtypes from different datasets using the Subclass Mapping (SubMap) methodology as previously described [14] (Gene Pattern Software, Version 3.0, Broad Institute, details in Data S1). This method relies on the principle of statistically assessed “enrichment” of the transcription program of one dataset, for marker gene lists derived from another dataset, as a function of relative differential ranking rather than absolute expression values, since the latter are platform and study-specific. “Mutual enrichment information” p values are generated to assess the “molecular match” between the different phenotypes and summarized in a Subclass Association Matrix. High mutual enrichment (low p value) indicates a strong molecular correspondence between subclasses in different datasets.

### Prediction of probability of oncogenic pathway activation or chemotherapy resistance in individual samples

We used publicly available and validated gene expression “read outs” of oncogenic pathway activation previously generated by experimentally controlled activation of these pathways *in vitro* [38]. Furthermore, we retrieved publicly available and validated gene expression models predicting the probability of resistance to individual chemotherapeutic agents that were generated using U133 Affymetrix array and drug response data from the NCI 60 cancer cell line panel [39], [40], [41]. Bayesian probit regression models estimating the probability of activation of each pathway or resistance to specific chemotherapeutics were trained in the experimental systems used to develop these signatures and applied on individual samples included in the Affymetrix oligonucleotide U133 datasets of our study. Gene expression models (“read outs”) of oncogenic pathway activation and chemoresistance are available at http://dig.genome.duke.edu/. Since these models were generated using oligonucleotide Affymetrix arrays and very few Affymetrix probesets were present in the cDNA datasets, our analysis was limited to the 3 Affymetrix oligonucleotide datasets of our study. Non-biological experimental variation between the *in vitro* arrays and the sarcoma datasets was corrected using a previously described batch effect adjustment algorithm [36]. Each individual sample was assigned a probability value (from 0 to 1) of pathway activation or resistance to a specific chemotherapeutic agent. A probability value higher than 0.5 was used as cut-off for predicted pathway activation.

Hierarchical clustering of samples based on their predicted probability values of oncogenic pathway activation was performed using the complete linkage algorithm with the Euclidean distance metric [37]. Non-parametric one-way Kruskal-Wallis and Mann-Whitney tests were applied to test whether the probability of oncogenic pathway activation and chemoresistance was different between different subtypes.

### MicroRNA gene-target enrichment analysis

To assess whether the gene expression patterns of STS samples were enriched for targets of microRNAs of interest, we used the functional class scoring method which tests the null hypothesis that the list of differentially expressed genes from each microRNA target set is a random selection from the entire project differentially expressed gene list, implemented in the NCI BRB Array Tools software, as previously described [42].

Additional details of the statistical methodology and bioinformatics algorithms described above are found in Data S1.

## Supporting Information

Data S1 Revised Supplementary Data File.
(0.05 MB DOC)

Figure S1 MFH-samples predicted as LIPO (MFH-LIPOs) cluster together with both myxoid and non-myxoid liposarcomas.
(0.27 MB TIF)

Figure S2 Complete Clustering Results.
(0.43 MB TIF)

Figure S3 Fold upregulation of selected genes associated with smooth muscle, fibroblast and adipocyte differentiation in MFH tumors predicted as leio-, lipo or fibrosarcoma respectively (compared to the rest of MFH tumors).
(0.19 MB TIF)

Table S1 Sensitivity and specificity of the 170-gene Nearest Centroid predictor for each STS class in the NCI, UK and JAPAN datasets (actual numbers of individual tumor subtypes are shown in parentheses).
(0.02 MB XLS)

Table S2 Sensitivity and specificity of the 170-gene Nearest Centroid predictor for each STS class in the STANFORD and MSKCC validation datasets.
(0.02 MB XLS)

Table S3 Genes included in 1st step predictor.
(0.04 MB XLS)

Table S4 Genes included in 2nd step predictor.
(0.02 MB XLS)

Table S5 Assessment of MFH reclassification across different datasets with the Sub Class Mapping methodology.
(0.02 MB XLS)

Table S6 Gene expression pattern of the Ras activated samples was enriched for targets of microRNAs of the let-7 family.
(0.02 MB XLS)

Table S7 Detailed clustering results of the classified MFH samples.
(0.02 MB XLS)

Table S8 Patterns of oncogenic pathway activation in different STS histologies.
(0.02 MB XLS)
