# Supplementary material for: Analysis of Multiple Sarcoma Expression Datasets: Implications for Classification, Oncogenic Pathway Activation and Chemotherapy Resistance
Source: PLoS One. 2010 Apr 1;5(4):e9747. doi: 10.1371/journal.pone.0009747 (PMC2848563; doi:10.1371/journal.pone.0009747)
Supplement: Data S1 — Revised Supplementary Data File. (0.05 MB DOC) [file pone.0009747.s001.doc]

**SUPPLEMENTARY MATERIALS AND METHODS**

**Validation of MFH reclassification across different datasets using Subclass Mapping (SubMap)**

Since hierarchical clustering cannot assess molecular correspondence between phenotypic classes present different datasets, we evaluated the molecular similarity between MFH samples (now reclassified into one of the 6 STS classes) and their corresponding STS samples from different datasets using the Subclass Mapping (SubMap) methodology ^1^ (as implemented in Gene Pattern Software, Version 3.0, Broad Institute, MIT).

In brief, Submap is an unsupervised subclass mapping method that identifies the correspondence or commonality of subtypes found in multiple, independent data sets potentially generated on different platforms. Suppose we have two independent datasets A and B with i and j candidate subclasses respectively (a subclass must contain at least 10% of the samples of a dataset to be considered as a candidate subclass). Marker gene lists ‘marker(Ai)’ for each candidate subclass in A (A1,…Ai) are determined based on the differential gene expression versus the rest of A subclasses, while genes in data set B are rank-ordered according to their correlation with each Bj subclass versus the other B subclasses to yield a gene list, ‘ranking(Bj)’. Association between Ai and Bj is evaluated by quantifying the over-representation of ‘marker(Ai)’ in the up-regulated end of the list ‘ranking(Bj)’ using Gene Set Enrichment Analysis (GSEA) as previously described ^2^. An enrichment score (ESAiBj) is calculated, and statistical significance is assessed as a p-value, pAiBj, by randomly permuting sample class labels in B and estimating the null distribution of ESAiBj score. This process is repeated by interchanging the role of A and B to compute ESBjAi and pBjAi. Mutual enrichment information is defined by combining pAiBj and pBjAi using the Fisher inverse chi-square statistic, Fij. Statistical significance is estimated based on a null distribution for the Fij generated by randomly picking the p from corresponding null distributions for ESAiBj and ESBjAi. A Bonferonni adjustment to account for multiple hypotheses testing is performed, and adjusted p-values are summarized in the subclass association matrix (SA matrix). A very low p value indicates a strong association between subclasses in different databases.

**Prediction of probability of oncogenic pathway activation or resistance to chemotherapeutic agents in individual STS samples**

We used signatures of experimentally controlled oncogenic pathway activation the majority of which are publicly available at <http://dig.genome.duke.edu/>. Furthermore, we retrieved publicly available gene expression models (available at http://dig.genome.duke.edu/) predicting the probability of resistance to individual chemotherapeutic agents that were generated using U133 Affymetrix array and drug response data from the NCI 60 cancer cell line panel. Both oncogenic pathway activation and chemotherapeutic response signatures have been validated in a variety of in vitro models and patient samples. Specific mathematical models based on Bayesian probit regression method estimating the probability of activation of each pathway or resistance to specific chemotherapeutics were trained in the experimental systems used to develop these signatures and applied on individual STS samples included in the Affymetrix U133 datasets of our study. To increase the confidence of the chemoresistance predictions, cell line samples of the NCI 60 panel that clustered falsely using the chemoresistance signatures, were excluded from the analysis. Thus, we included 25, 16 and 11 cell lines from the NCI-60 panel for the adriamycin, cyclophosphamide and docetaxel predictions. Non-biological experimental variation between the experimental system arrays and the sarcoma datasets was corrected using a previously described batch effect adjustment algorithm ^3^. Each individual STS sample was assigned a probability value of pathway activation or resistance to a specific chemotherapeutic agent, from 0 to 1. A probability value higher than 0.5 was used as cut off for pathway activation or resistance to a specific chemotherapeutic agent.

**MicroRNA gene target enrichment analysis**

To assess whether the gene expression patterns of STS samples predicted to have activated Ras pathway were enriched for targets of microRNAs – each taken as a functional unit – of the let-7 family, the functional class scoring method was used. This analysis was repeated in the three U133 datasets of our study. All predefined target genelists of the 8 microRNAs of the let-7 family (let-7a,b,c,d,e,f,g,i) (available at miRBase, Sanger Bioinformatics Institute, <http://microrna.sanger.ac.uk/sequences/>) were analyzed for differential expression between STS samples predicted to have Ras activation versus the rest ^4^. The statistical significance for differential expression of each microRNA target genelist was estimated using the functional class scoring method ^5^. In brief, a p value was computed for each gene in each microRNA target genelist and then the set of p-values for each genelist was summarized by the LS score (mean negative natural logarithm of the p-values of the respective single gene univariate test) and the Kolmogorov-Smirnov (KS) score. For each microRNA genelist, significance was assessed by testing the null hypothesis that the list of differentially expressed genes from each list was a random selection from the entire project gene list. N genes (equal to the number of the target genes of each microRNA) were randomly selected from the project gene list, and the LS and KS statistics and their random distribution were computed (100,000 random selections). The LS (KS) permutation p-value was defined as the proportion of random simulations for which the LS (KS) statistic was larger than the LS (KS) statistic computed for the microRNA target genelist. Statistical significance was set at 0.005.

**REFERENCES**

1. Hoshida Y, Brunet JP, Tamayo P, et al: Subclass mapping: identifying common subtypes in independent disease data sets. PLoS ONE 2:e1195, 2007

2. Subramanian A, Tamayo P, Mootha VK, et al: Gene set enrichment analysis: a knowledge-based approach for interpreting genome-wide expression profiles. Proc Natl Acad Sci U S A 102:15545-50, 2005

3. Johnson WE, Li C, Rabinovic A: Adjusting batch effects in microarray expression data using empirical Bayes methods. Biostatistics 8:118-27, 2007

4. Ambros V: The functions of animal microRNAs. Nature 431:350-5, 2004

5. Pavlidis P, Qin J, Arango V, et al: Using the gene ontology for microarray data mining: a comparison of methods and application to age effects in human prefrontal cortex. Neurochem Res 29:1213-22, 2004

6. Baird K, Davis S, Antonescu CR, et al: Gene expression profiling of human sarcomas: insights into sarcoma biology. Cancer Res 65:9226-35, 2005

7. Nielsen TO, West RB, Linn SC, et al: Molecular characterisation of soft tissue tumours: a gene expression study. Lancet 359:1301-7, 2002

8. Henderson SR, Guiliano D, Presneau N, et al: A molecular map of mesenchymal tumors. Genome Biol 6:R76, 2005

9. Nakayama R, Nemoto T, Takahashi H, et al: Gene expression analysis of soft tissue sarcomas: characterization and reclassification of malignant fibrous histiocytoma. Mod Pathol 20:749-59, 2007

10. Detwiller KY, Fernando NT, Segal NH, et al: Analysis of hypoxia-related gene expression in sarcomas and effect of hypoxia on RNA interference of vascular endothelial cell growth factor A. Cancer Res 65:5881-9, 2005
