# Supplementary figures and images for: Analysis of Multiple Sarcoma Expression Datasets: Implications for Classification, Oncogenic Pathway Activation and Chemotherapy Resistance
Source: PLoS One. 2010 Apr 1;5(4):e9747. doi: 10.1371/journal.pone.0009747 (PMC2848563; doi:10.1371/journal.pone.0009747)

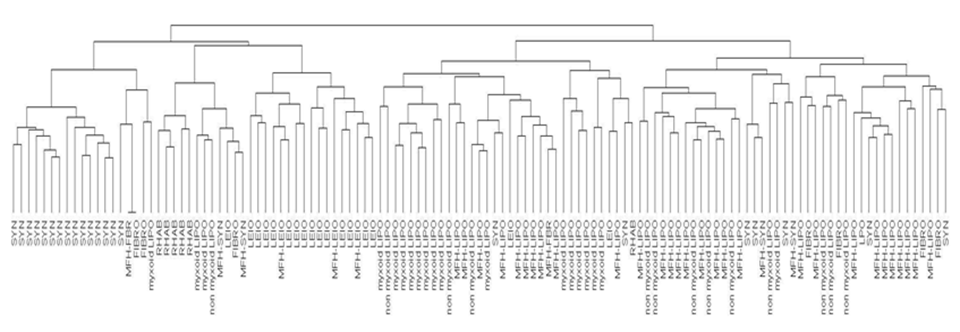

Supplement: Figure S1 — MFH-samples predicted as LIPO (MFH-LIPOs) cluster together with both myxoid and non-myxoid liposarcomas. (0.27 MB TIF) [file pone.0009747.s002.tif]

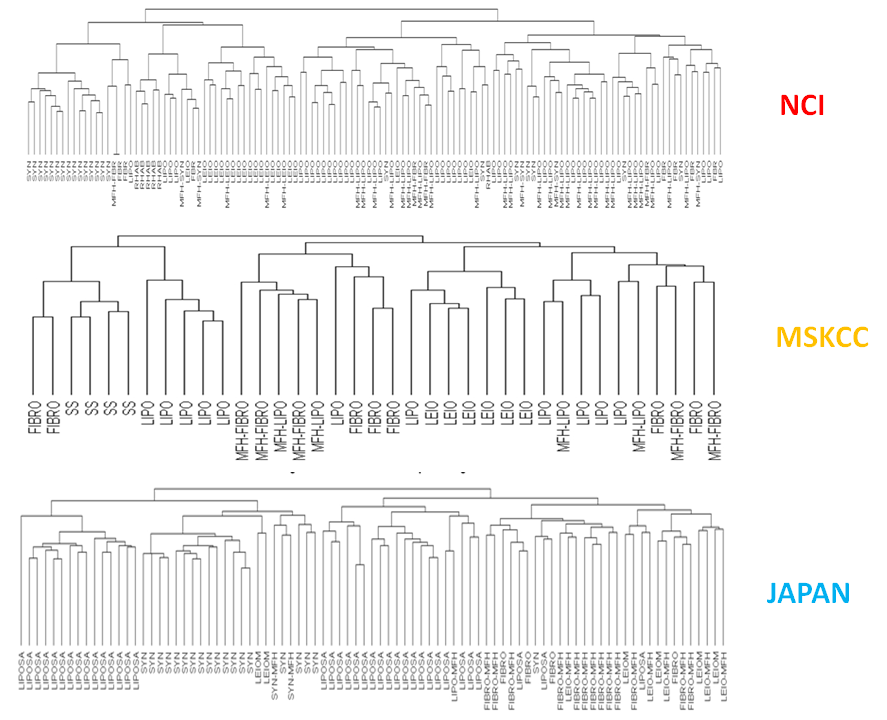

Supplement: Figure S2 — Complete Clustering Results. (0.43 MB TIF) [file pone.0009747.s003.tif]

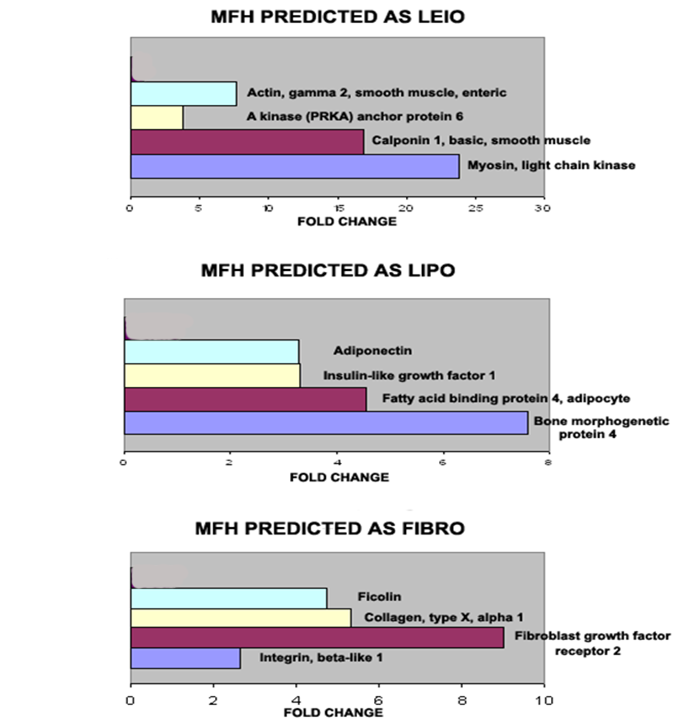

Supplement: Figure S3 — Fold upregulation of selected genes associated with smooth muscle, fibroblast and adipocyte differentiation in MFH tumors predicted as leio-, lipo or fibrosarcoma respectively (compared to the rest of MFH tumors). (0.19 MB TIF) [file pone.0009747.s004.tif]
